# Streaming histogram sketching for rapid microbiome analytics

**DOI:** 10.1186/s40168-019-0653-2

**Published:** 2019-03-16

**Authors:** Will PM Rowe, Anna Paola Carrieri, Cristina Alcon-Giner, Shabhonam Caim, Alex Shaw, Kathleen Sim, J. Simon Kroll, Lindsay J. Hall, Edward O. Pyzer-Knapp, Martyn D. Winn

**Affiliations:** 1 Scientific Computing Department, STFC Daresbury Laboratory, Warrington, UK; 2 IBM Research, The Hartree Centre, Warrington, UK; 3 Quadram Institute Bioscience, Norwich Research Park, Norwich, UK; 4 Department of Medicine, Section of Paediatrics, Imperial College London, London, UK

## Abstract

**Background:**

The growth in publically available microbiome data in recent years has yielded an invaluable resource for genomic research, allowing for the design of new studies, augmentation of novel datasets and reanalysis of published works. This vast amount of microbiome data, as well as the widespread proliferation of microbiome research and the looming era of clinical metagenomics, means there is an urgent need to develop analytics that can process huge amounts of data in a short amount of time.

To address this need, we propose a new method for tyrhe compact representation of microbiome sequencing data using similarity-preserving sketches of streaming k-mer spectra. These sketches allow for dissimilarity estimation, rapid microbiome catalogue searching and classification of microbiome samples in near real time.

**Results:**

We apply streaming histogram sketching to microbiome samples as a form of dimensionality reduction, creating a compressed ‘histosketch’ that can efficiently represent microbiome k-mer spectra. Using public microbiome datasets, we show that histosketches can be clustered by sample type using the pairwise Jaccard similarity estimation, consequently allowing for rapid microbiome similarity searches via a locality sensitive hashing indexing scheme.

Furthermore, we use a ‘real life’ example to show that histosketches can train machine learning classifiers to accurately label microbiome samples. Specifically, using a collection of 108 novel microbiome samples from a cohort of premature neonates, we trained and tested a random forest classifier that could accurately predict whether the neonate had received antibiotic treatment (97% accuracy, 96% precision) and could subsequently be used to classify microbiome data streams in less than 3 s.

**Conclusions:**

Our method offers a new approach to rapidly process microbiome data streams, allowing samples to be rapidly clustered, indexed and classified. We also provide our implementation, Histosketching Using Little K-mers (HULK), which can histosketch a typical 2 GB microbiome in 50 s on a standard laptop using four cores, with the sketch occupying 3000 bytes of disk space. (https://github.com/will-rowe/hulk).

## Background

The global corpus of microbiome sequence data is being augmented daily with vast volumes of data, particularly as a result of large-scale sequencing initiatives such as the Human Microbiome Project (HMP) [1], the Earth Microbiome Project [2] and the Global Ocean Survey [3]. Data outputs will continue to increase, particularly as metagenomics within the clinical field is more widely being accepted and adopted [4], and as sequencing costs continue to decline [5].

We are now at the point where our ability to analyse microbiome data quickly and effectively is the main bottleneck in our workflows, particularly when it comes to real-time sequencing platforms [5, 6]. In addition, we also need to ensure that existing microbiome data remains accessible and usable (including for end users, e.g. clinicians), so that it can be readily incorporated into our new analyses and generate testable hypotheses for validation/confirmation in experimental systems. It is becoming clear that current microbiome analytics are not suitable in this age of ‘big data’, particularly in terms of data retrieval and sample classification [7].

Current microbiome analytics can be largely split into referenced-based or de novo approaches [8]. Whereas reference-based analyses (such as taxonomic classification) can often result in sequencing data being excluded and high computational requirements, de novo approaches circumvent these issues. For example, the pairwise comparison of k-mer spectra is a de novo analysis method that has been routinely used in recent years for clustering microbiomes using dissimilarity measures [9, 10] (see Table 1 for a summary of technical terms). These measures are used to identify microbiome composition changes in studies that involve longitudinal sampling or multiple isolation sites [11]. However, k-mer spectra can still take considerable time to compute and are relatively large in file size, and new sample comparisons require additional computation. As well as this, machine learning (ML) frameworks will struggle to use these de novo outputs as feature vectors due to their scale. This is a potential barrier to the use of these methods in microbiome analytics as ML can help solve many of the data problems encountered in genomics and holds great potential for microbiome analytics [12].

**Table 1:** Summary of technical terms

Term	Definition
Consistent weighted sampling	An efficient method of sub-sampling histogram data that takes into account the frequency of each bin
De novo	Analyses based solely on the collected sequence data
Dimensionality reduction	Representing the sequence data in a metagenome by a relatively small number of collective quantities
Dissimilarity measure	A measure of how dissimilar two metagenomes are, typically used to identify significant changes in microbiome composition
Feature vectors	A set of key quantities of a dataset that can be used as input to a machine learning algorithm
Histosketch	A small approximate representation of histogram data, such as a k-mer spectrum.
Jaccard similarity	A measure of the similarity of two datasets based on the proportion of shared members.
K-mer	A short sub-sequence extracted from a read or genome
K-mer spectrum	The set of all observed k-mers, together with their abundances in the sequence dataset
Locality-sensitive hashing	A method of dimensionality reduction which hashes sequence data in such a way that similar sequences are kept together
Reference-based	Making use of existing reference genomes to align and classify new sequencing data

The application of other techniques to reduce dimensionality or complexity of genomic data has tried to address some of these issues. By reducing the dimensionality of data, these techniques offer approximate answers to bioinformatic questions (within definable error bounds) but can obtain these answers with much reduced time and memory requirements. These techniques have ranged from distributed string mining of informative k-mers [13] to the recent use of locality-sensitive hashing (LSH) [14–18]. MinHash is one form of LSH that has greatly improved genomic analysis speeds for operations such as sample clustering, database searching and phylogenetic estimation; it works through reducing sequence data to small, representative sketches using a set of minimum k-mer hash values [14]. However, although MinHash-based tools can be used to great effect for certain microbiome analytics (e.g. what genomes are in my microbiome?), there remain limitations to standard MinHash techniques, such as the loss of k-mer frequency information and the impact of relative set size on the Jaccard similarity estimates [19, 20]. Although some MinHash genomic implementations address these limitations (e.g. the over-sketching and track-abundance methods of the MinHash tools ‘finch’ and ‘sourmash’), they do not utilise the frequencies of all observed k-mers in generating the sketch for a given sample. With this in mind, we suggest that additional de novo microbiome analysis methods are required in this era of microbiome ‘big data’ in order to perform essential tasks such as rapid similarity, indexing and classification operations. This is particularly pertinent within a clinical metagenomics setting, as accurate and ‘useful’ data is required for downstream analysis and clinical decision making, e.g. antibiotic treatment choices [7]. This paper offers a novel method to augment existing microbiome analysis tools and workflows, with a view to mitigating the above limitations.

Here we present a data sketching method for clustering, indexing and classifying microbiome sequencing data. We also describe and demonstrate our software implementation, Histosketching Using Little K-mers (HULK), that is a user-friendly and efficient implementation of the method. Our method reduces microbiome sequence data streams to an updateable ‘histosketch’ of the underlying k-mer spectrum for a sample. We utilise consistent weighted sampling to incorporate k-mer frequency information into the histosketch, allowing the use of weighted and standard Jaccard similarity for histosketch comparisons and sample retrieval [21]. Our method combines the recently proposed histogram sketching algorithm of Yang et al. with count-min sketching of k-mer spectra and our recent implementation of LSH forest indexing for microbiome searching [17, 22, 23]. We show our method to accurately cluster microbiome samples by sample type and demonstrate the utility of these histosketches to create and search microbiome sequence databases. Finally, we show that histosketches are suitable features for training ML classifiers and can accurately classify microbiome samples according to antibiotic treatment history in at-risk preterm infant populations. We anticipate that our method and accompanying software will work toward addressing the current demand for fast and accurate microbiome comparisons in temporal and spatial studies.

## Materials and methods

**Fig. 1:**
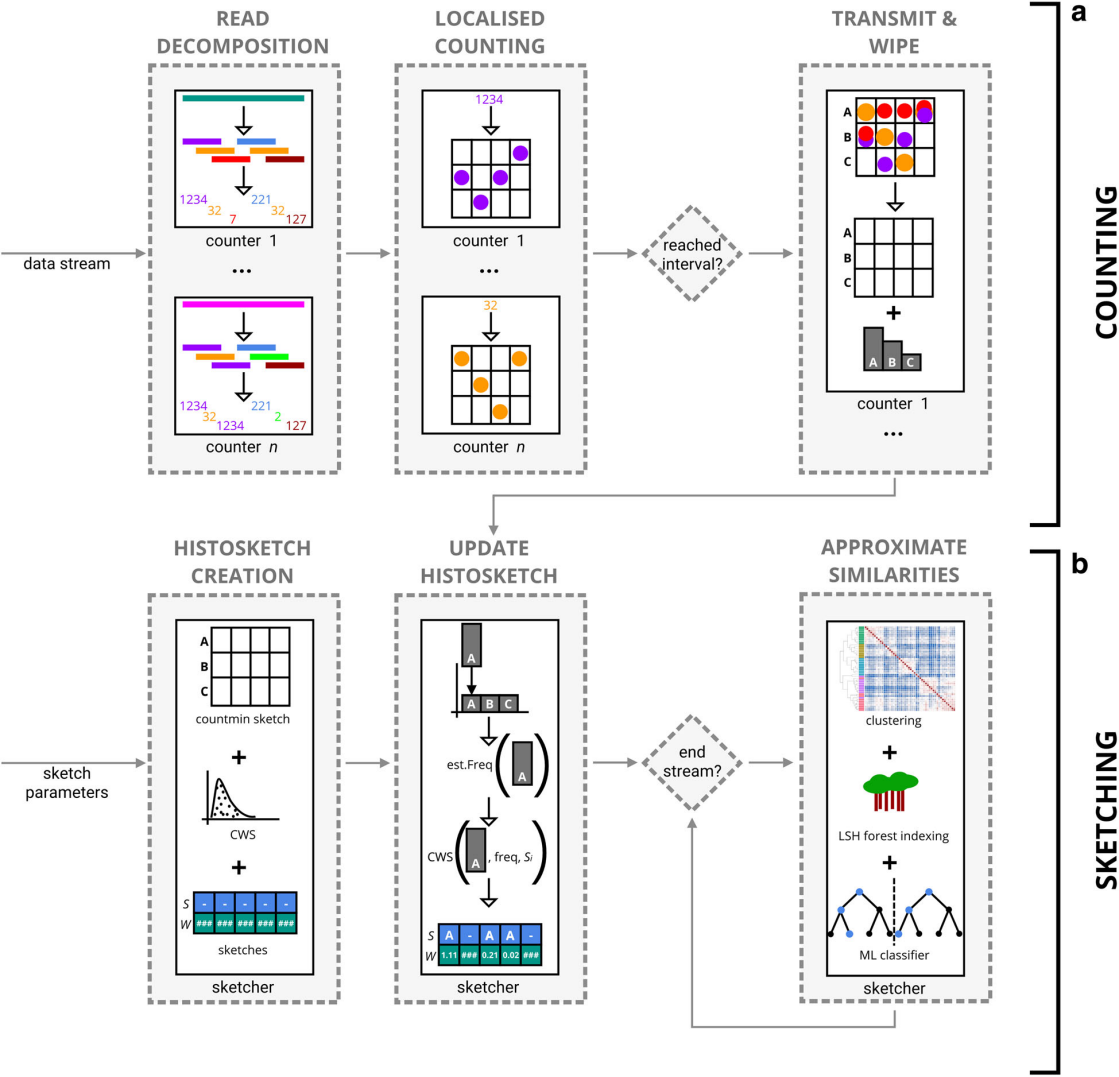
Overview of our method to histosketch microbiome samples from sequence data streams. **a** During counting, sequence reads are collected from the data stream by *n* counting processes. Reads are decomposed to canonical k-mers, encoded to uint64 values and used to increment local count-min sketches. Once *X* reads have been received from the data stream, approximate k-mer counts from the counting processes are transmitted as histogram elements to the single sketching process. **b** To update the histosketch, the incoming histogram element is hashed and compared against each hash value (*W*) or the previous histosketch (*S*), updating *S* and *W* if a new minimum is encountered. To hash the incoming vector, uniform scaling is applied and a cumulative frequency estimate is made using a count-min sketch; we then utilise CWS to generate a hash value for the updated histogram bin

Here we describe our method for the compact representation of microbiome sequencing data using similarity-preserving histosketches of streaming k-mer spectra (Fig. 1). We then document our implementation, HULK, and describe several use cases.

### Histosketching microbiome data

We use the k-mer spectrum (a normalised vector of k-mer frequencies) to represent microbiome diversity, which is a standard analysis method that allows for metagenome dissimilarity analysis [9, 10]. However, rather than computing and storing a full k-mer spectrum after reading the sequence data, which is resource intensive (in terms of memory or disk space), we use the recently proposed histosketch data structure to maintain a set of fixed size sketches to approximate the overall k-mer spectrum as it is received from a data stream [22]. The histosketch has two properties making it suitable for this application: (1) it is updateable and (2) it is similarity-preserving. Thus, as new data is received, we can incrementally update the histosketch of the underlying k-mer spectrum and also approximate similarity to other spectra.

We view the k-mer spectrum as a histogram, where k-mers from a microbiome sample are hashed uniformly across N bins and the frequency value of a bin corresponds to observed k-mer frequency. In order to incorporate both the bin and frequency (a weighted set) into the histosketch, we employ consistent weighted sampling (CWS) to generate hash values for each histogram element, which ensures that the computational complexity of hashing is independent of bin frequency [21, 22].

#### Consistent weighted sampling

As highlighted in the introduction, a drawback to the efficient set similarity estimations afforded by MinHash sketches is that the input is restricted to binary sets and does not account for weighted sets (e.g. k-mer frequencies). To overcome this, histosketching employs CWS to account for element frequency and approximate the generalised Jaccard similarity between weighted sets, without splitting each weighted element into sub-elements and computing independent hash values (quantization) [20, 21, 24, 25].

For a set of k-mer spectrum histogram bins, *W*, where each bin, *k*, has a frequency value, *W*_*k*_ ≥ 0, CWS will produce a sample, (*k*, *a*_*k*_): 0 ≤ *a*_*k*_ ≤ *W*_*k*_, which is both uniform and consistent. This CWS sample (*k*, *a*_k_) corresponds to the k-mer spectrum histogram bin (*k*) and its scaled weight (*a*_*k*_). The CWS sample is uniformly sampled from *∪*_*k*_ {*k*} × [0, *W*_*k*_], meaning that the probability of selecting *k* from *W* is proportional to the k-mer frequency, *W*_*k*_, and *y* is uniformly distributed on [0, *W*_*k*_]. The sample is also consistent as given two weighted sets, *W*1 and *W*2, if ∀*k*, *W*1_*k*_ ≤ *W*2_*k*_, a sub-element (*k*, *a*_*k*_) is selected from W1 and satisfies *y*_*k*_ ≤ *W*2_*k*_, then (*k*, *a*_*k*_) will also be selected from *W*2 [20, 21].

In order to generate a consistent sample for a member of a weighted set, CWS first samples three distributions using all elements from the set. So, for each *k* in *W*, CWS samples from *r*_*k*_ ∼ gamma (1, 2), *β*_*k*_ ∼ uniform (0, 1) and *c*_*k*_ ∼ gamma (1, 2). Once these distributions have been sampled, CWS can then generate a consistent sample for any given element from the set. CWS uses the following two equations (Eqs. 1 and 2) to output a consistent weight, *a*_*k*_.

1 $$ {y}_k=\exp\;\left(\log\;{W}_k-{r}_k{\beta}_k\right) $$

2 $$ {a}_k=\frac{c_k}{y_k\exp \left({r}_k\right)} $$

Equations 1 and 2 generate ‘active indices’ and are used to hash an element (*k*) in proportion to its weight (*W*_*k*_). The two active indices allow for the implicit construction of an exponential distribution for each weighted element (in our case, a k-mer spectrum histogram bin and its frequency). In the context of histosketching, hashing a histogram bin is performed by drawing a value from the exponential distribution parameterized by the bin frequency, meaning that a minimum hash value for a histogram bin will be sampled in proportion to the frequency of that bin. Use of the log domain in the active indices avoids most transcendental function computations.

#### Histosketch creation

Equations 1 and 2 describe the CWS method, which we apply to sample a k-mer spectrum in a way that takes the relative abundance of k-mers into account. To generate a sketch of a k-mer spectrum originating from a biological sample, the k-mer spectrum is sampled *Z* times, where *Z* is the size of the sketch.

We will denote our underlying k-mer spectrum (a histogram) as *V*, with cardinality |*V*| = *X* (*i* = 1, …, *X*). The corresponding histosketch we will denote as *S*, with cardinality |*S*| = *Z* (*j* = 1, …, *Z*). To initialise *S* from *V*, the first three independent variables are sampled from the CWS distributions: *r*_*i*_,_*j*_ ∼ gamma (1, 2), *c*_*i*_,_*j*_ ∼ gamma (1, 2) and *β*_*i*_,_*j*_ ∼ uniform (0, 1) for *i* = 1, …, *X* and *j* = 1, …, *Z*. We then use Algorithm 1 of Yang et al. for histosketch creation [22]. The sketch, *S*, and the corresponding hash values, *A*, are both kept as the histosketch (*A* allows for incremental sketch updating).

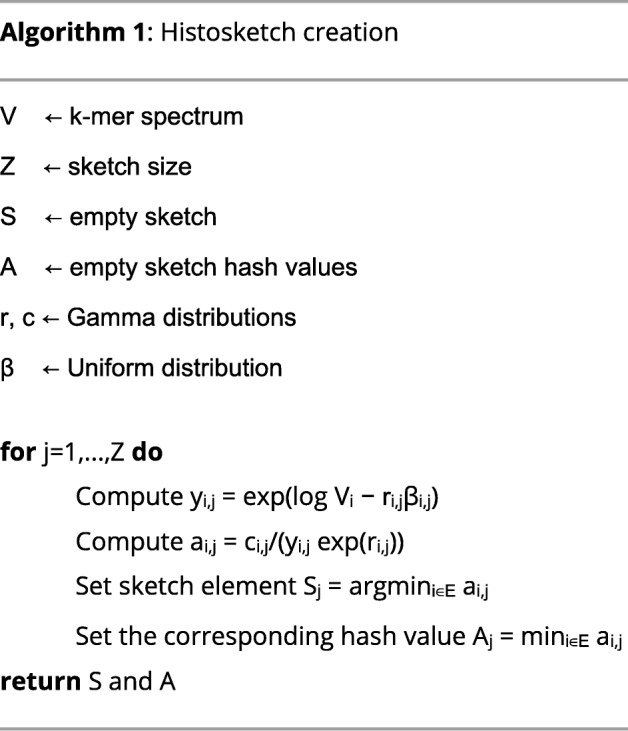



To summarise, to create an element *S*_*j*_ for one histosketch slot, based on underlying histogram *V*, we select the histogram element *V*_*i*_ whose hash value is minimal and also keep the corresponding hash value (*A*_*j*_).

#### Histosketch updating

To update the histosketch as a new histogram element is received, the previous sketch *S* and the sketch hash values *A* are required. In its simplest form, the histosketch incremental update works by hashing and evaluating the incoming element against each slot of the histosketch. The cumulative bin frequency of the incoming element is estimated using a persistent count-min sketch [26]; the frequency estimate is then used to update the hash value for the required histogram bin. If this hash value is now a minimum, the sketch slot and corresponding hash value are updated.

In addition to this histosketch update method, we can also utilise the gradual forgetting weights of the original histosketch implementation to adjust for changes in the underlying distribution (concept drift) [22, 27]. Prior to the update, uniform scaling is applied to the estimate frequency counts. After this, the histosketh hashes are scaled using a decay weight before evaluating against the incoming element.

### Our implementation

We have implemented our method as an easy to use a program called HULK. HULK is written in Go (version 1.11) and compiles for a variety of operating systems and architectures. It is also packaged for installation with Bioconda and Biocontainers [28, 29]. The HULK software uses a UKRI licence, which is free for academic use. HULK utilises a concurrent pipeline pattern that is driven by the flow of data between structs. This pattern facilitates the streaming of data from the standard input (STDIN), as well as from disk, and allows the HULK subcommands to be piped together and operate on data streams:

$$ \mathrm{gunzip}-\mathrm{c}\ \mathrm{reads}.\mathrm{fq}\mid \mathrm{hulk}\ \mathrm{sketch} $$

#### Histosketching

The HULK subcommand ‘sketch’ performs histosketching on a FASTQ data stream. Reads are collected from the data stream by one or more independent counting processes (Fig. 1: counting), each utilising a separate Go routine for concurrent counting. Each counting process will count reads until an interval is reached (e.g. 1 million reads have been seen) or a signal is sent (e.g. the sample has been classified using a downstream ML classifier, see the “Random forest classifier” section). The counting processes will then send their count data via a Go channel to be histosketched and then wipe their stores before collecting more reads.

A read is received by the counting process as a slice of bytes, and the canonical k-mers are encoded to unsigned integers (uint64) using bit shift operations. Once encoded, the k-mer frequency is updated in the local store of the counting process. To ensure the counting processes operate in a fixed amount of memory, we again use the count-min sketch data structure to record frequency estimates for the k-mer spectrum [23, 26]. The count-min sketch counters are used as a proxy for the number of bins in the underlying k-mer spectrum. The relative accuracy of the count-min sketch is within a factor of epsilon, with probability delta. The default values of epsilon and delta are 0.0001 and 0.9, respectively, resulting in count-min sketch dimensions of four hashtables, each with 20,000 slots. Using these defaults, the maximum resident set size of the HULK sketching process on one CPU is an average of 1 .5Mb.

Once an interval is reached, the counting processes each send their k-mer spectrum data in a randomised order to the single histosketching process; this process follows the incremental histosketch update process described above (Fig. 1: sketching).

#### Distance estimation

HULK includes two distance subcommands, ‘distance’ and ‘smash’. Running ‘hulk distance’ will run a pairwise comparison of two histosketches and output the Jaccard, weighted Jaccard, Bray Curtis or Euclidean metrics. Running `hulk smash` will perform a pairwise comparison of two or more histosketches and output a matrix of Jaccard or weighted Jaccard similarities:

$$ \mathrm{hulk}\ \mathrm{smash}--\mathrm{wjsMatrix}-\mathrm{d}./\mathrm{dir}-\mathrm{with}-\mathrm{sketches} $$

The calculation of weighted Jaccard distance utilises the histosketch bin and corresponding hash values; Eq. 3 shows the calculation of the weighted Jaccard distance for two histosketches, *S* and *T*.

3 $$ \mathrm{weightedJaccardDistance}\left(S,T\right)=1-\frac{\cap {\sum}_k\min \left({S}_k,{T}_k\right)}{\cup {\sum}_k\max \left({S}_k,{T}_k\right)} $$

#### Indexing

HULK utilises the LSH forest self-tuning indexing scheme as employed in our previous work [17]. Briefly, this scheme will take a query and return a subset of nearest-neighbour candidates, based on the number of hash collisions [30]. The two parameters to tune this index are the number of hash functions to encode an item (K), and the number of hash tables to split an item across (L). To tune index prior to adding items, multiple combinations of *K* and *L* are evaluated by false positive/negative rate at the given Jaccard similarity threshold. To add a histosketch to the index, we use only the sketch *S* (i.e. not the hash values *A*, see the “Histosketch creation” section); the sketch is split into *L* equally sized chunks of *K* hashes. The chunks are hashed to a binary string (little-endian ordering) and stored in the corresponding hash table. Prior to searching the index, the hash tables are transferred to a set of arrays and sorted.

The HULK index operations are performed using the ‘index’ subcommand. Three modes are available: create, add and search. To create an index, the LSH forest index is initialised using a Jaccard similarity and error rate thresholds, and then each histosketch is split into the appropriate number of chunks and added as described in the above paragraph. The index is written to disk in the unsorted form.

$$ \mathrm{hulk}\ \mathrm{index}-\mathrm{r}\ \mathrm{create}-\mathrm{n}\ \mathrm{a}.\mathrm{index}-\mathrm{j}\ 0.90-\mathrm{d}./\mathrm{ref}-\mathrm{sketches}--\mathrm{r}\mathrm{ecursive} $$

To add a histosketch to an existing index, the index is loaded and the histosketch is added using the existing index parameters. To search the index, the index is first loaded and the hash tables are transferred to a set of arrays and sorted. The query set of histosketches are then queried in series, and the similar histosketches are returned (by label) that are within the Jaccard similarity threshold that was set during indexing.

$$ \mathrm{hulk}\ \mathrm{index}-\mathrm{r}\ \mathrm{search}-\mathrm{n}\ \mathrm{a}.\mathrm{index}-\mathrm{j}\ 0.90-\mathrm{d}./\mathrm{query}-\mathrm{sketches}--\mathrm{r}\mathrm{ecursive} $$

#### Random forest classifier

We implemented a random forest classifier (RFC) as an example ML classifier to showcase the applicability of our histosketches as features for predicting microbiome sample labels. Our implementation (BANNER) is written in Python (version 3.6) and is distributed with HULK, as well as through Bioconda and Pypi. Source code is available at https://github.com/will-rowe/banner. It uses the SciKit Learn (version 0.19.2) implementation of the RFC [31]. Again, we use only the sketch values *S* and discard the hash values *A*. BANNER trains on 80% of the available data using bootstrapping and 1000 estimators; testing then uses the remaining 20% of the available data and does this with tenfold cross-validation. Once trained, the RFC model is serialised. To classify histosketches with BANNER, the RFC model is first loaded and un-serialised, before collecting histosketches from STDIN, allowing the output of `hulk sketch` to be piped so that histosketches can be classified as they are generated:

$$ \mathrm{hulk}\ \mathrm{sketch}-\mathrm{f}\ \mathrm{sample}.\mathrm{fastq}--\mathrm{stream}-\mathrm{p}\ 8\mid \mathrm{banner}\ \mathrm{predict}-\mathrm{m}\ \mathrm{banner}.\mathrm{rfc} $$

The predict subcommand will only terminate once it makes a prediction above a set probability threshold or the sketching processes finishes.

### Evaluating performance

The full commands and code to evaluate the performance of our implementation can be found in the HULK repository (https://github.com/will-rowe/hulk/tree/master/paper). HULK version 0.0.2 was used in all experiments (release 0.0.2, commit 97ba8ac).

For running the clustering and indexing experiments, the simulated short reads from the Critical Assessment of Metagenome Interpretation (CAMI) project (dataset to benchmark new programs against highly complex and realistic metagenomic datasets) were downloaded in FASTQ format [32]. For each complete read set, HULK sketches (k-mer size = 21, histosketch size = 512), sourmash (version 2.0.0a11) sketches (k-mer size = 21, sketch size = 512, track abundance = true) and Simka (version 1.4.0) k-mer spectra (k-mer size = 21) were created and pairwise Jaccard distances were loaded into Python (version 3.6.5) using Pandas (version 0.23.4) [33] and clustered using Seaborn (version 0.9.0) (clustering method = complete). For running HULK and Simka, both were restricted to 12 CPUs per FASTQ file and run using LSF on a high performance computing cluster (Atos Bull Sequana, Intel Skylake nodes).

As an additional clustering experiment, we used a recently published dog microbiome dataset to detect dietary intervention using histosketches on varying levels of sequencing data (ENA: PRJEB20308) [34]. This study reported a significant shift in the taxonomic composition of dog microbiomes when diets were changed from a baseline diet. The full dataset contains 1.9 terabasepairs of sequencing data, of which we sampled 0.005%, 0.05% and 0.5% of each microbiome. We histosketched these samples (k-mer size = 21, histosketch size = 512) and clustered them as above.

For performing the RFC analysis, an RFC model was constructed as described in the “Indexing” section, using a clinically relevant dataset: gut microbiome profiles from a cohort of healthy preterm from a single hospital. This is part of a wider neonate clinical study that is longitudinally profiling their gut microbiome and correlating their findings to health outcomes and antibiotic prescription. Faecal samples from preterm infants were collected and their bacterial DNA extracted following the protocols described by Alcon-Giner et al. [35]. Shotgun metagenomic libraries were prepared from 500 ng of genomic DNA which was sheared into fragments of ~ 450 bp. The sheared DNA was purified and concentrated using an SPRI-clean-up kit. Library construction entailed an end repair, A-tailing and adapter ligation steps. Following adapter ligation, samples were amplified and indexed by PCR using established Illumina paired-end protocols. A portion of each library was used to create an equimolar pool, and pooled libraries were subjected to 125 bp paired-end sequencing on a HiSeq 2500 V4. The cohort was labelled according to whether the infants were receiving prophylactic antibiotic treatment or no antibiotics. The histosketches from 108 FASTQ files (BioProject: PRJEB28428) were split into training (80%) and testing (20%) groups. When using the RFC model to classify the incremental sketch updates of blinded samples, HULK was run using sketching intervals of 10,000, 100,000 and 1,000,000 reads using a 4 core laptop (k-mer size = 7, histosketch size = 42, concept drift decay ratio = 0.02).

## Results

The results presented here evaluate our implementation of histosketching for rapid microbiome comparisons, in terms of both the accuracy of the tool and its potential applications. All analyses can be re-run using the analysis workbooks (https://github.com/will-rowe/hulk/tree/master/paper/analysis-notebooks).

### Clustering microbiome datasets

**Fig. 2:**
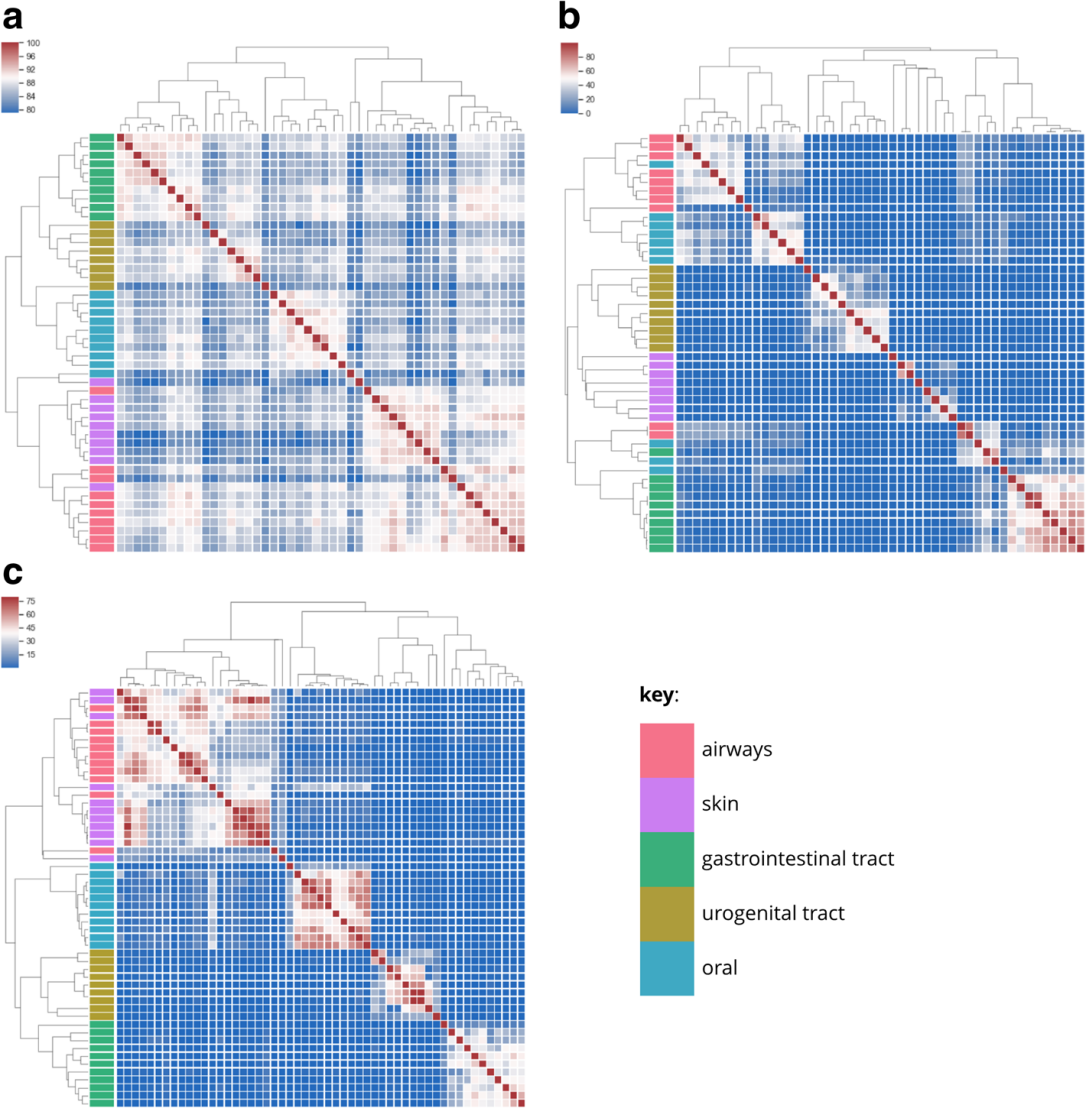
Hierarchical clustering of CAMI short read microbiome samples [32]. Heatmaps show the pairwise Jaccard similarity between microbiome samples (ranging from 0% (blue) to 100% (red)); colormap ranges are computed using robust quantiles and dendrogram clades are coloured by body site. **a** HULK histosketches (k-mer size = 21, histosketch size = 512) for 48 microbiome samples were sketched in 1 min 30 s (12 cores per histosketch). **b** sourmash MinHash sketches (k-mer size = 21, sketch size = 512, track abundance = true) for 48 samples were sketched in 25 min 17 s. **c** Simka k-mer spectra (k-mer size = 21) for 48 microbiome samples were computed in 24 min 1 s (12 cores per spectrum)

We begin by assessing the speed and ability of HULK to cluster metagenomes based on pairwise similarities, and compare to the performance of two other popular methods. The CAMI metagenome sequence data for 48 microbiome samples were sketched by HULK in 1 min 30 s and by sourmash in 25 min 17 s, and the full k-mer spectra were computed by Simka in 24 min and 1 s. The combination of sketching and a parallel implementation thus makes HULK significantly faster than the other methods tried. Hierarchical clustering identified five distinct groups using both the HULK histosketches (Fig. 2a) and the full k-mer spectrum of Simka (Fig. 2c); these groups corresponded to the five body sites of the CAMI project (denoted by the coloured bars on the dendrograms). The hierarchical clustering of the sourmash minhash sketches resulted in six groups (Fig. 2b). Using the HULK sketches, two samples failed to cluster by body site (skin and airways), whereas three samples failed to cluster for the Simka full k-mer spectra (skin and airways) and 8 samples failed to cluster correctly for the sourmash sketches (skin, airways and oral).

**Fig. 3:**
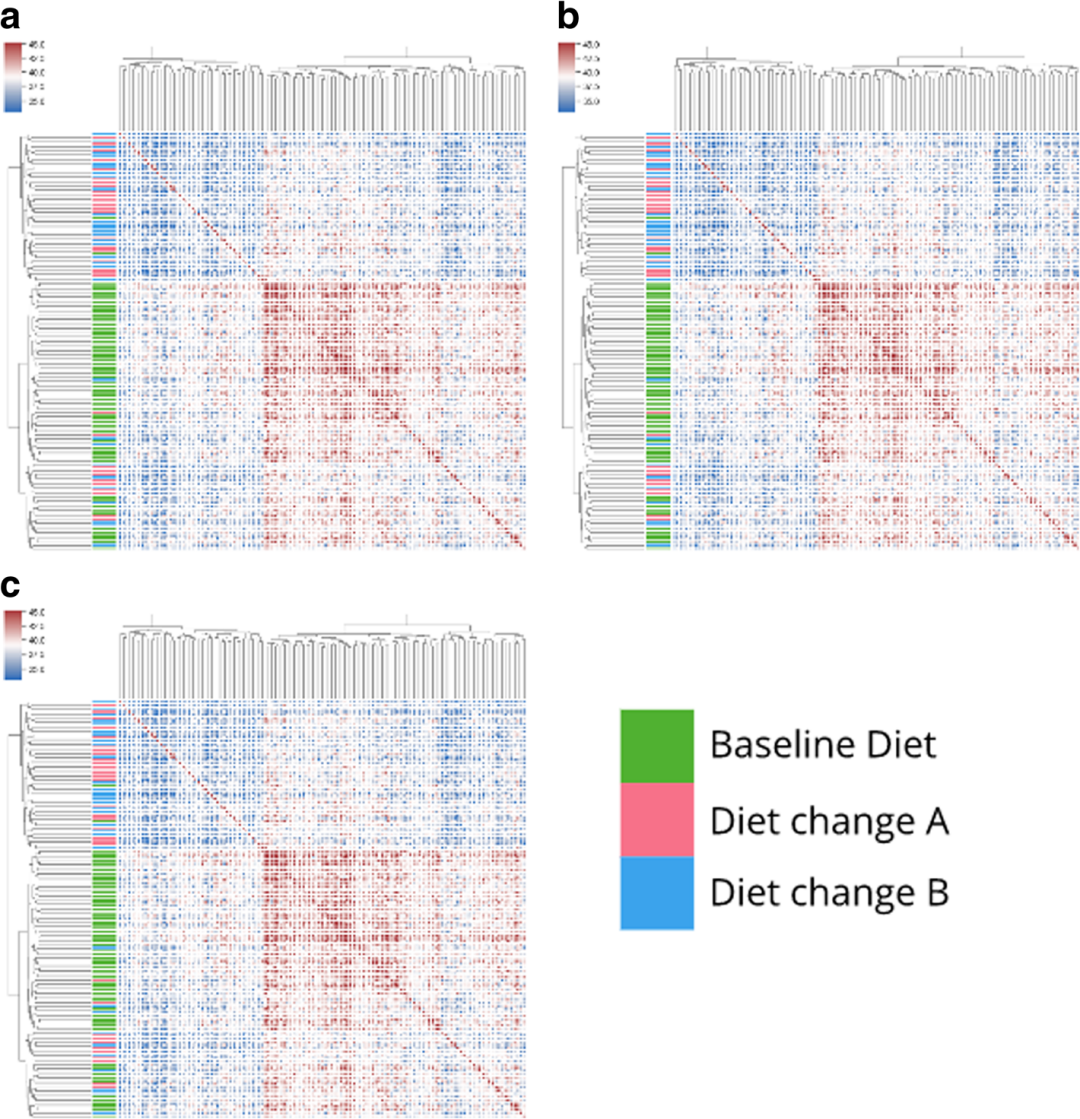
Hierarchical clustering of dog microbiome samples [34]. **a**, **b** and **c** correspond to clustered histosketches from 0.005%, 0.05% and 0.5% of sample reads, respectively. Heatmaps show the pairwise Jaccard similarity between microbiome samples (ranging from 0% (blue) to 100% (red)); colormap ranges are computed using robust quantiles and dendrogram clades are coloured by diet. The majority of microbiome samples from the dogs on the baseline diet clustered together (green); however, the samples taken after these dogs were put on to an altered diet (pink/blue) and did not show any distinct clustering pattern

To show the ability of our method to cluster incomplete data streams in a biological meaningful way, we performed incremental histosketch updating on data streams from a collection of dog microbiome samples. As the data was downloading, we histosketched the data stream (using fastq-dump to stream the download); approximately 0.005%, 0.05% and 0.5% of the reads from each sample (129 samples total) were processed and then clustered based on pairwise Jaccard similarity (Fig. 3). At all intervals, we found a clear separation of histosketches between microbiome samples from dogs receiving the baseline diet and those receiving an altered diet (high/low protein). This is in agreement with the findings of the original study, where they reported a significant shift in the taxonomic composition of dog microbiomes when diets were changed [34]. The total microbiome data for the original study was stored in 3096 runs across 129 samples, amounting to 1.9 terabasepairs. Complete download of this dataset from the ENA took over 7 days using fastq-dump with 20 parallel downloads. Sketching the initial 0.005% of the data stream took an average of 4 s per sequencing run (approximately 100 s per sample).

### Indexing microbiome collections

**Fig. 4:**
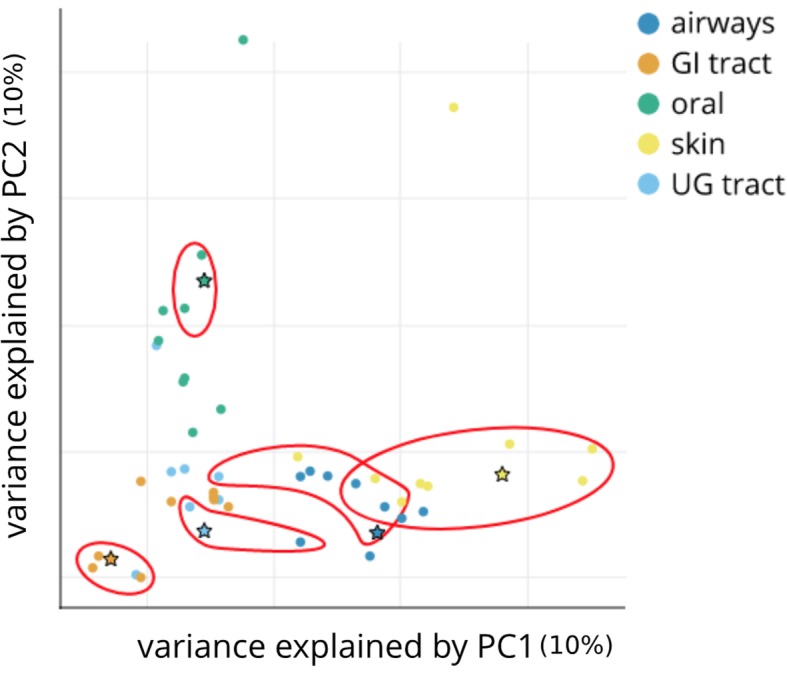
Principal component analysis of histosketches from CAMI short read microbiomes, with the 48 samples coloured by body site [32]. Circular data points indicate the histosketches used to build the LSH forest index and stars data points indicate histoketches used as search queries. Red rings enclose the returned LSH Forest search results for each search query (Jaccard similarity threshold > 90%)

We next test the LSH forest self-tuning indexing scheme as applied to HULK histosketches. The histosketches from the CAMI metagenome sequence data were labelled by body site before one sample was randomly removed from each group and used as a search query. The remaining sketches were indexed using HULK in 0.039 s (with a Jaccard similarity threshold of 0.90). Each query histosketch returned a subset of CAMI samples, at least one of which was from the same body site (Fig. 4). The oral query returned only oral samples; the gastrointestinal (GI) tract, airways and skin queries returned predominantly samples from their own respective body sites, whilst the urogenital (UG) tract returned one sample from the same body site, plus another from airways. When overlaid on principal components 1 and 2 of a PCA analysis, the search queries are grouped nearest their respective LSH forest search results (Fig. 4).

### Classifying microbiomes using machine learning

Finally, we wanted to determine how the above approaches could be used to profile clinically relevant datasets, providing key data that could be used in a healthcare setting. Thus, we trained a random forest classifier using a microbiome collection that included gut microbiome profiles from a cohort of healthy preterm neonates from the St Mary’s Hospital, NICU, London, and labelled the samples according to whether the infants were receiving prophylactic antibiotic treatment or no antibiotics. The accuracy on the test set during RFC construction was 0.97, with an F1 score of 0.96. When histosketching entire FASTQ files from the blinded microbiome samples from the cohort, histosketches were successfully classified using the previously trained RFC as being from an antibiotic treated neonate (classification probability = 0.99, average runtime = 28.38 s) (Table 2). Additionally, when streaming reads and performing incremental histosketch updates, classifications could be made using the incrementally updated histosketches in 1.91 s (sampling interval = 10,000 reads, classification probability = 0.82) and go on to achieve classifications with probability ≥ 0.90 after two histosketch updates (average runtime = 2.09 s) (Table 2). Once classified at a probability ≥ 0.90, the data stream for a sample was terminated and a new sample data stream was then histosketched.

**Table 2:** Average random forest classification runtimes for predicting antibiotic vs. no-antibiotic treated neonatal microbiomes using read sampling intervals and concept drift (probability threshold = 0.9, *k* = 7, *s* = 42, decay ratio = 0.02, *p* = 8)

Sampling interval (no. reads)	Runtime to reach initial classification (seconds)	Initial classification probability	Runtime to reach ≥ 0.9 classification probability (seconds)	Sampling intervals to reach ≥ 0.9 classification probability
No interval	28.38	0.99	28.38	na
1,000,000	9.16	0.96	9.16	1
100,000	2.08	0.87	2.09	2
10,000	1.91	0.82	2.17	4

*na* not applicable

## Discussion

In this paper, we have presented a new method, as well as several practical examples, for rapid microbiome analytics using streaming histogram sketching. This work has been in direct response to the call for improved microbiome analytics in this era of big data, massive microbiome sequencing initiatives and the realistic prospect of clinical metagenomics [4, 7]. We feel that our microbiome sketching method and the applications shown here go toward addressing this challenge.

As outlined in the introduction, the dimensionality reduction methods that have only recently been applied to genomics have been a great advance toward the goal of rapid microbiome analytics, facilitating fast similarity queries such as identifying genomes or genes within metagenome samples [16, 17]. Our dimensionality reduction method for the comparison, indexing and classification of microbiomes offers a novel and complementary method to these existing ones. In particular, it addresses the main limitations of traditional MinHash for certain microbiome analyses. These being: (1) histogram sketching is not impacted by mismatched set size [19] and (2) histogram sketching accounts for weighted sets (e.g. k-mer frequency).

Whilst our method is designed for microbiome comparison studies, it should be noted that it is based upon the assumption that the microbiomes being compared have shared membership, i.e. differences between samples will primarily be in taxa abundance, rather than membership. Consequently, our method (as with other k-mer spectra methods) will perform best with temporal and spatial microbiome samples. Where studies have microbiome samples that do not share substantial levels of similarity in their base constituents (e.g. samples from different environments), other sketching methods would be better suited for microbiome analytics. On a similar note, we want to also state that HULK was not designed for bacterial isolate comparisons/microbiome membership queries (due to the infrequent use of k-mer spectrum comparisons in this area), and direct the reader to other tools that excel in this area (such as microbiome screening offered by Mash/sourmash etc.).

In terms of the advantages of HULK over other de novo analysis methods (e.g. k-mer spectra dissimilarity analysis), we have shown here that the computation of histogram sketches from complete metagenomic datasets is 16 times faster than the computation of the full k-mer spectra and 17 times faster than the computation of the MinHash sketch (see the “Clustering microbiome datasets” section and Fig. 2). In terms of performing sketching faster than sourmash’s compute function, we should stress that the better performance seen by HULK is due to the ability to parallelise the histosketching process (both tools run in a similar time on a single core). All tools produced a fairly accurate clustering of the microbiome samples, according to the body site from which the microbiomes were designed to originate, with HULK actually producing the most accurate clustering in this sense. We suggest that the incorporation of k-mer frequency information into the sketch generation may account for the better clustering performance of HULK compared to that of sourmash, which differs by using k-mer abundance for weighting the distance calculations and not the sketch generation. As well as faster analysis times, histogram sketching has a much smaller footprint as the entire k-mer spectrum does not need to be kept in memory or written to disk, and the resulting sketches are much smaller in size than the full spectrum. As with other sketching methods, HULK also does not require re-computation of previously sketched samples in order to make new comparisons (provided new samples are sketched using the same parameters).

In addition, we showed that histosketching microbiome samples can work on incomplete data streams and allow samples to be clustered by the underlying microbiome composition when using just a small proportion of the total reads (see the “Clustering microbiome datasets” section and Fig. 3). Our results suggest that only a small proportion of the total data stream needs to be sampled in order to cluster the samples according to a particular treatment using histosketch similarity. Although we managed to identify the time point when the diet was changed from baseline to an altered diet, we were not able to differentiate between the two altered diets using our sketches from the initial data stream. This may be due to insufficient sampling of the data stream; however, the original study did not report being able to differentiate between the two altered diets either (see Fig. 2c, Coelho et al. [34]).

In the “Indexing microbiome collections” and “Classifying microbiomes using machine learning” sections, we demonstrated that microbiome histosketches can be efficiently indexed and also used as features in ML classification, which are both typically hard to do using the full k-mer spectra due to their scale and sparsity [7]. In terms of the LSH forest index for microbiome sample retrieval, our results showed that a histosketch from a given body site would predominantly return microbiome samples from the same body site (Fig. 4). Only the oral histosketch query returned solely oral samples, which is likely due to the high similarity observed between these datasets (Fig. 2). On the whole, these results indicate that histosketches of k-mer spectra can offer an efficient and fast way to index and query collections of microbiome data.

Our performance evaluation of HULK using an RFC illustrates how incremental sketching (as highlighted in Fig. 3) can be combined with ML in order to classify a microbiome and stop processing a data stream (see the “Classifying microbiomes using machine learning” sections). The RFC experiment showed that histosketches can be generated using part of a data stream more quickly than those generated using the full dataset, at the expense of classification probability. Also, by varying the sampling interval (i.e. number of reads), different classification probabilities are obtained; a histosketch generated at a given sampling interval may need to receive several updates before it meets a probability threshold. As such, there is a balance between sampling interval and the resulting classification probability. Therefore, the use of sampling intervals may be useful for obtaining quick, approximate results, but for greater confidence in microbiome classifications, sampling interval may need to be increased at the expense of runtime.

This demonstration of classifying partial data streams is a step forward in dealing with streaming genomics data; the combination of incremental histosketch updates with a ML classifier (and associated classification probabilities) allows for the possibility of terminating data streams in applications such as real-time sequencing [14]. Here, we used this approach to quickly evaluate longitudinal samples from a cohort, identifying whether there is a response to a specific treatment. In this case, we have used this sketching approach to differentiate between those preterm infants that had received antibiotics, versus those that did not. This is important clinically as antibiotic treatment in preterm infants and is associated with significant alterations in the gut microbiota, which may link to increase risk of development serious conditions such as necrotising enterocolitis or sepsis [35–37]. Thus, a rapid and discriminatory microbiome profiling method for this fragile and at-risk patient cohort, or indeed for other clinical microbiome samples, could prove useful for intervention or treatment options. Alternatively, it could be applied to real-time sequencing platforms and inform the sequencer when enough data has been produced. These examples illustrate how this method could be used in the coming era of clinical metagenomics [4]. We are not restricted to using RFC and it would be very useful to evaluate other more sophisticated ML approaches that can utilise histosketches as feature vectors, as well as determining the impact of larger microbiome sample collections on classification accuracy. Indeed, in our recent study evaluating ML for microbiome classification, we showed that histosketches generated from the HMP metagenome collection (~ 670 samples) can be used with a variety of ML classifiers (RVMs, SVMs, RFCs, NBCs), with support-vector machines performing better than RFs in some cases [38]. As well as this, we could refine our ML models further by identifying the more significant elements of the histosketch in terms of their influence over the model training. This in turn could reveal more information relating to the underlying k-mer spectrum of a sample, which may be of use in downstream applications (e.g. feature extraction).

Despite the promise shown by our use of histosketching and ML to evaluate and terminate a stream of sequencing data, it should be noted that we currently have no guarantees in our method that the input FASTQ data is randomly ordered, and this in turn could impact the robustness of the resulting sketches. For instance, known biases in the Illumina sequencing platform can result in tiles or edges of the flow cell that produce reads of lower quality, which will impact the histosketch. We have attempted to mitigate some of these sequence quality issues by the inclusion of an optional read quality trimming algorithm in the HULK sketch command.

For future work into microbiome analytics, the histogram sketching method presented has potential for further refinement and improvements in order meet the big data challenges that microbiome research presents. Of these, we have already identified that further work into the use of histosketches in ML is definitely needed, particularly with the hope of improving classification accuracy and expanding out from the binary classification task we have shown here. In addition, we would like to further explore the idea of concept drift for gradually forgetting outdated histogram elements [22]. We included concept drift in our ML classification experiment using incrementally updated histosketches and observed increasing classification probability with histosketch updates (see the “Classifying microbiomes using machine learning” sections). We envisage that this could be useful to experiment further in terms of real-time sequencing applications. For instance, histosketching with concept drift may be useful in a clinical setting when, combined with environmental sensors, surface microbiomes could be continually monitored and any changes in microbiome composition reported to then be checked for presence of pathogens.

Finally, we have shown that microbiome samples can be histosketched on a laptop with a few cores and a small, fixed amount of memory. In order to fully take advantage of this performance, histosketching needs to move beyond command line interfaces. To this end, we have begun working on a WebAssembly (WASM) port of HULK to enable client side sketching (WASM available Go Version 1.11) so that users can histosketch their own microbiome data and compare just the sketches against online databases, ensuring their microbiome data remains private but enabling quick and easy microbiome analytics.

## Conclusions

Histosketching generates compact representations of microbiomes from data streams, facilitating sample indexing, similarity-search queries, clustering, and the application of machine learning methods to analyse microbiome samples in the context of the global microbiome corpus.
